# Collagen mimetic peptide repair of the corneal nerve bed in a mouse model of dry eye disease

**DOI:** 10.3389/fnins.2023.1148950

**Published:** 2023-05-16

**Authors:** Lauren K. Wareham, Joseph M. Holden, Olivia L. Bossardet, Robert O. Baratta, Brian J. Del Buono, Eric Schlumpf, David J. Calkins

**Affiliations:** ^1^Department of Ophthalmology and Visual Sciences, Vanderbilt Eye Institute, Vanderbilt University Medical Center, Nashville, TN, United States; ^2^Stuart Therapeutics, Inc., Stuart, FL, United States

**Keywords:** dry eye, ocular collagen, collagen mimetic peptides (CMPs), neuropathy, collagen reparative, extracellular matrix

## Abstract

The intraepithelial sub-basal nerve plexus of the cornea is characterized by a central swirl of nerve processes that terminate between the apical cells of the epithelium. This plexus is a critical component of maintaining homeostatic function of the ocular surface. The cornea contains a high concentration of collagen, which is susceptible to damage in conditions such as neuropathic pain, neurotrophic keratitis, and dry eye disease. Here we tested whether topical application of a collagen mimetic peptide (CMP) is efficacious in repairing the corneal sub-basal nerve plexus in a mouse model of ocular surface desiccation. We induced corneal tear film reduction, epithelial damage, and nerve bed degradation through a combination of environmental and pharmaceutical (atropine) desiccation. Mice were subjected to desiccating air flow and bilateral topical application of 1% atropine solution (4× daily) for 2 weeks. During the latter half of this exposure, mice received topical vehicle [phosphate buffered saline (PBS)] or CMP [200 μm (Pro-Pro-Gly)_7_, 10 μl] once daily, 2 h prior to the first atropine treatment for that day. After euthanasia, cornea were labeled with antibodies against βIII tubulin to visualize and quantify changes to the nerve bed. For mice receiving vehicle only, the two-week desiccation regimen reduced neuronal coverage of the central sub-basal plexus and epithelial terminals compared to naïve, with some corneas demonstrating complete degeneration of nerve beds. Accordingly, both sub-basal and epithelial βIII tubulin-labeled processes demonstrated increased fragmentation, indicative of nerve disassembly. Treatment with CMP significantly reduced nerve fragmentation, expanded both sub-basal and epithelial neuronal coverage compared to vehicle controls, and improved corneal epithelium integrity, tear film production, and corneal sensitivity. Together, these results indicate that topical CMP significantly counters neurodegeneration characteristic of corneal surface desiccation. Repairing underlying collagen in conditions that damage the ocular surface could represent a novel therapeutic avenue in treating a broad spectrum of diseases or injury.

## Introduction

Innervation of the cornea is the densest in the human body (Zander and Weddell, 1951; Schimmelpfennig, 1982). Corneal nerves comprise both a sparse autonomic component and a far more abundant sensory component (Marfurt et al., 1989, 2010). The autonomic component represents some 10–15% of corneal nerve fibers and is nearly entirely sympathetic, involving axons from the superior cervical ganglion (Marfurt et al., 1989). These course through the ciliary nerves to form the limbal plexus (Al-Aqaba et al., 2019). Neurons that mediate sensory innervation of the cornea largely express two primary peptides, calcitonin gene–related peptide (CGRP) and substance P (Muller et al., 2003; He and Bazan, 2016). These send axons through the ophthalmic branch (V_1_) of the trigeminal nerve to a sparse population of neurons in the trigeminal ganglion (Belmonte et al., 2017). The corneal nerve bed arises from large bundles of fibers entering the stroma at the limbus. These ramify extensively throughout the epithelium to form the intraepithelial corneal nerves (Stepp et al., 2020). As these converge centripetally, they form the tell-tale swirl pattern that also marks the pattern of corneal epithelial cells migrating from the limbus toward the apex of the cornea (Marfurt et al., 2010; Al-Aqaba et al., 2019). These fibers end as intraepithelial corneal nerve terminals that ramify in and between the apical cells of the epithelium. Thus, the basic structure of the nerve bed taken as a whole comprises two primary components that arise from the large stromal bundles penetrating at the limbus: the sub-basal, intraepithelial plexus, and the superficial terminals that characterize their endings (Marfurt et al., 2010; Al-Aqaba et al., 2019). Importantly, this basic architecture is conserved between human and mouse cornea (He and Bazan, 2016).

The dense corneal nerve plexus is critical to maintaining homeostatic function of the ocular surface (Asiedu, 2022; Vereertbrugghen and Galletti, 2022). Maintenance of a healthy tear film prevents surface desiccation and relies upon neurosensory information from the cornea that is essential for adequate blinking and tearing. The corneal nerve plexus also contributes to immune regulation of the ocular surface and to integrity of the corneal epithelial layer (Galletti and de Paiva, 2021; Asiedu, 2022; Wu et al., 2022). Damage to the corneal nerve bed can lead to enhanced epithelial permeability and diminished capacity for epithelial repair (Beuerman and Schimmelpfennig, 1980), while intact CGRP signaling within the nerve bed is essential to re-epithelialization (Mikulec and Tanelian, 1996; Hwang et al., 2021; Asiedu et al., 2022a). Nerve bed damage is endemic to dry eye disease (DED) (McKay et al., 2019; Guerrero-Moreno et al., 2020), which is the most prevalent progressive ocular surface disease, afflicting many millions of people worldwide (Belmonte et al., 2017; Farrand et al., 2017). Dysfunctional sensory nerves in dry eye exacerbate progression through reduced basal tearing and blinking and leads to neuropathic pain (Dieckmann et al., 2017; Vereertbrugghen and Galletti, 2022). Exposure to environmental desiccation in mice reduces sub-basal corneal nerve density and increases pro-inflammatory dendritic cells (Simsek et al., 2018). This effect has been demonstrated in human studies in systemic conditions such as diabetic chronic kidney disease and rheumatoid arthritis (Asiedu et al., 2022b; Bitirgen et al., 2023). Conversely, higher corneal nerve density in certain mouse strains is related to more efficient repair of wounds to the corneal epithelial layer (Pham et al., 2019).

Collagen accounts for some 90% of corneal thickness, distributing broadly across the various layers (Meek, 2009). Recently we hypothesized that a novel approach to repair corneal collagen, which is normally slowly replaced by mesenchymal and other cells, could offer a new therapeutic avenue to reduce ocular surface damage, inflammation, and neuropathic pain in DED (Baratta et al., 2022). In support of this idea, application of synthesized collagen peptides in animal models of dry eye disease promote corneal tear adherence and facilitate epithelium stabilization (Lee et al., 2017). Collagen mimetic peptides (CMPs) directly repair damaged collagen by intercalating into and reforming fragmented triple helices (Chattopadhyay et al., 2012, 2016; Chattopadhyay and Raines, 2014). Finally, we demonstrated that a type I collagen mimetic (CMP 03A) restored the corneal epithelium following acute damage (Baratta et al., 2021, 2022).

Therapies such as CMPs that restore the extracellular matrix (ECM) also have therapeutic potential by promoting repair of neurons (Ren et al., 2015; Song and Dityatev, 2018). We showed that intraocular delivery of a CMP (CMP 03A) protected and repaired neurons in both chronic and acute injury models of the visual system (Ribeiro et al., 2022). Here, we test whether topical application of this same CMP was efficacious in reducing damage to the ocular surface of the mouse eye induced by a combination of environmental and pharmaceutical (atropine) desiccation. We find that topical application of CMP improved integrity of the corneal epithelium, tear film production, and corneal sensitivity instigated by a two-week desiccation regimen compared to vehicle controls. CMP also prevented neuronal degeneration by reducing fragmentation and enhancing both sub-basal and epithelial neuronal coverage compared to vehicle controls. Together, our results indicate that topical CMP treatment reduces the neurodegenerative characteristic of corneal surface desiccation and that repairing underlying collagen in conditions that damage the ocular surface could represent a novel therapeutic avenue in treating ocular surface diseases.

## Materials and methods

### Animals

All experiments and procedures were conducted in accordance with the Association for Research in Vision and Ophthalmology (ARVO) statement for the use of animals in ophthalmic and vision research and were approved by the Vanderbilt University Institutional Animal Care and Use Committee. Mice were housed in a facility managed by Vanderbilt University Division of Animal Care, with *ad libitum* access to water and standard mouse chow and a 12 h light cycle (lights on at 6:30 a.m. and off at 6:30 p.m.). For all experiments, adult male and female C57/B6/J mice aged 8 weeks were obtained from Charles River Laboratory (Wilmington, MA, USA).

### Corneal desiccation regimen

**FIGURE 1:**
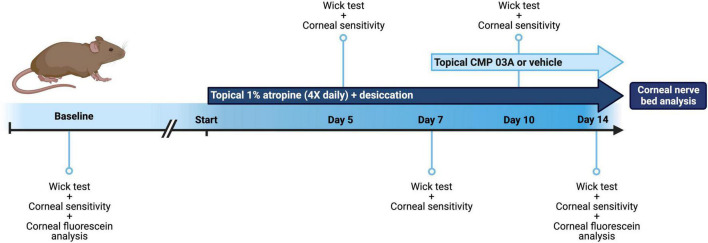
Schematic of dry eye desiccation model and treatment regimen. Baseline tear film production (wick test) and corneal sensitivity measurements were taken in all mice before commencement of the study (baseline = day 0). Animals were then treated bilaterally with topical 1% atropine 4× daily with desiccation for a total of 7 days. At day 5 and day 7, tear film and corneal sensitivity measurements were taken. At day 7, in addition to atropine + desiccation, mice were then treated topically with application of vehicle or CMP 03A (200 μm), 1× per day bilaterally for a further 7 days. Tear film production and corneal sensitivity measurements were carried out at day 10 and upon completion of the study at day 14. *In vivo* corneal fluorescein analysis was completed at baseline (day 0) and on day 14, after which mice from all cohorts were euthanized and corneal tissue processed for analysis.

We modified a protocol designed for desiccation of the rabbit eye (Burgalassi et al., 1999). Mice were housed in standard cages in a light- and temperature-controlled room at 19 ± 1°C and 44 ± 4°C humidity. We prepared 1% atropine solution by dissolving 10 mg of atropine sulfate (Millipore Sigma, Burlington, MA, USA) in 1 ml 1× phosphate buffered saline (PBS). The solution was filter sterilized using a 0.22 μm filter (Millipore Sigma, Burlington, MA, USA) and stored at 4°C for no more than 3 days. During preliminary studies, we found that atropine alone for 2 weeks did reduce tear film production in mice but did not cause significant corneal nerve bed damage after 2 weeks. Thus, mice were randomized prior to baseline measurements and were then subjected to desiccating air turbulence using a fan directed into the cage for 9 h daily from 9 a.m. to 6 p.m. During hours of air turbulence, 4 μL of a 1% atropine solution was applied topically to the corneal surface at regular intervals for a total of 4 applications to both eyes daily. After 6 p.m., mice were returned to normal air flow and atropine dosage was stopped until 9 a.m. the following day. For atropine application, mice were anesthetized with 2% isoflurane in oxygen for 30 s for drop application and then incubated in the presence of 2% isoflurane for a further 30 s to increase atropine penetration. The regimen of combined desiccation and atropine application was continued for 14 days (Figure 1).

After baseline tear film wick test and corneal sensitivity measurements were taken, mice were subjected to the cornea desiccation protocol for 7 days. At day 5 and day 7, tear film wick test and corneal sensitivity measurements were taken (Figure 1). To assess whether CMP 03A [(Pro-Pro-Gly)_7_] treatment prevented nerve damage induced by the desiccation regimen, at day 7 mice were randomly assigned to receive either vehicle or CMP bilaterally for an additional 7 days. Vehicle or CMP 03A was applied once daily via a 10 μL topical drop at least 1 h prior to the first atropine treatment for that day. Tear film wick test and corneal sensitivity measurements were taken at day 10 and day 14. Animals were euthanized at the end of day 14. For these experiments, *N* = 11–29 (vehicle), 11 (CMP 03A), and 13 (naïve), with additional animals added to the vehicle group from unrelated, parallel experiments.

### Tear film wick test

Tear film production was measured via wick test using endodontic absorbent paper points (EAPP, World Precision Instrument, Sarasota, FL, USA) as previously described (Kilic and Kulualp, 2016). Awake animals were scruffed, and a single EAPP was placed using forceps into the lower lid of the right eye. The EAPP was left in place for 1 min before tear absorption was measured along the wick using a standard ruler. Tear production was measured in each animal at baseline, at 5 and 7 days prior to vehicle or CMP treatment, and again at day 10 and 14 prior to euthanasia.

### Corneal sensitivity measurements

Corneal sensitivity was measured on the central cornea using a Cochet-Bonnet aesthesiometer (Luneau Ophtalmologie, Chartres Cedex, France) using a modified protocol previously described (Edwards et al., 2017; Stepp et al., 2018; Pham et al., 2019). The length of the monofilament was manually varied, and the following lengths were tested: 3.5, 2.5, 2, 1.5, 1- and 0.5-mm in each animal. Awake animals were scruffed, and the monofilament touched perpendicular to the central cornea 6 times at each length. A positive response was recorded if blinks occurred at a frequency of 50% (i.e., 3 blinks per a total of 6 taps) and the monofilament length was noted as the sensitivity threshold measurement. If no blink response could be elicited at a monofilament length of 0.5 cm then the sensitivity threshold was recorded as 0.

### Corneal fluorescein staining

Corneal fluorescein staining was performed at baseline (day 0, i.e., naïve) and at the conclusion of the study (day 14). Mice were anesthetized with intraperitoneal injection of ketamine (135 mg/kg) and xylazine (7.5 mg/kg). Eyes were washed gently with sterile 1× PBS to remove surface debris. A 2 μl drop of a filtered 1% sodium fluorescein solution was then added to the surface of the cornea. The fluorescein solution was left for 1 min before excess solution was removed with a Q-tip at the outer cornea of the eye. The mouse was then placed under a Nikon AZ100 fluorescent microscope for imaging. Fluorescence images taken in the FITC channel using a 0.5× objective with 7× zoom and a 1 s exposure time.

### Immunohistochemistry

Immediately after euthanasia, fresh cornea were dissected and placed into 4% paraformaldehyde (PFA) solution for 15 min at room temperature. Cornea were washed once in 1× PBS and then transferred to a 20% sucrose solution at room temperature for 30 min, and then into a 30% sucrose overnight at 4°C. The following day, cornea were subjected to 3 sets of 5 min freeze-thaw cycles before washing in 1× PBS. Cornea were then placed in a 1% Triton X-100 in 1× PBS solution for 3 h, shaking at room temperature. Cornea were blocked in a solution containing 2% normal donkey serum (NDS; 017-000-121, Jackson ImmunoResearch Laboratories, Inc., West Grove, PA, USA), 2% bovine serum albumin (BSA) and 1% Triton X-100/1× PBS for 2 h, shaking at room temperature. After blocking, corneas were placed in primary antibody solution (2% NDS/0.2% Triton X-100 in 1× PBS) with antibody [1:500 mouse anti-βIII tubulin (MAB5564, Millipore Sigma, Burlington, MA, USA)] for 3 days, shaking at 4°C. After 3 days of primary antibody incubation, cornea were washed 3× for 10 min per wash in 0.2% Triton X-100/1× PBS solution. Cornea were then placed in secondary antibody solution (1% NDS/0.2% Triton X-100 in 1× PBS) containing secondary antibody [Donkey anti-mouse Alexa Fluor-488 (715-546-150, Jackson ImmunoResearch Laboratories, Inc., West Grove, PA, USA)] for 2 h, shaking at room temperature. Cornea were then washed 3× in 1× PBS and mounted in DAPI Fluoromount-G (0100-20, SouthernBiotech, Birmingham, AL, USA) for imaging.

### Corneal tissue fluorescent imaging

Fluorescent whole corneal images were taken using a Nikon Ni fluorescent microscope and a 20× objective; whole corneal images were taken en montage. Central corneal stacked images were acquired using an Olympus FV-1000 inverted confocal microscope using a 40× oil objective.

### Corneal nerve density and fragmentation analysis

Images were acquired in z-stacks through the entire central cornea using an Olympus FV1000 confocal microscope at 800 px^2^ × 800 px^2^ resolution on a 40× objective. Due to the curvature of the flattened cornea, different regions of the same corneal image had sub-basal nerves and epithelial nerve endings in different z-planes and thus needed to be stacked separately to compose the flattened final image. To aid in segmentation, a Python script was utilized to split the image into nine smaller z-stacked tiles that could be processed individually. The bounds for each tile sub-stack were manually chosen then flattened using the standard deviation z-stacking method in ImageJ. This produced a single stacked image each for the sub-basal plexus and epithelial terminal plexus. Each image was manually thresholded in ImageJ and the binary image saved. The python script re-stitched the binarized, flattened smaller tiles to give final stacked images of both the sub-basal nerve plexus and epithelial endings. The complete binarized images were then processed using a Python script that performed a flood-fill algorithm on every contiguous region, or fragment, identified in the binary image. Each unique, contiguous fragment was randomly assigned a red-greenblue (RGB) value, and an image generated to visually assess nerve fragmentation. The number of unique RGB values generated in this way was saved as a record of the number of fragments in each image. Fragmentation was then quantified as the number of fragments per image normalized to nerve coverage (area of binary image with nerve labeling).

### Epithelial DAPI fluorescence intensity analysis

In ImageJ, stacked confocal images representing total central corneal epithelium were converted to 8-bit and a Gaussian filter applied to reduce speckle noise. Intensity line plots at 5 fixed, evenly spaced *x*-coordinates, and 5 fixed, evenly spaced *y*-coordinates were plotted; a total of 10 line plots across each image per animal were measured. Raw intensity line plots for each group were averaged and the first-derivative function plotted using GraphPad Prism version 9.0 (GraphPad Software, San Diego, CA, USA). Area under the curve (AUC) and number of peaks were generated using GraphPad from the first derivative curves in each group.

### Statistical analysis

All data are presented as mean ± standard error of the mean (SEM) unless otherwise stated. Statistical analyses were performed, and graphs made using GraphPad Prism version 9.0 (GraphPad Software, San Diego, CA, USA) and SigmaPlot 15.0 (Systat-InPixon, Palo Alto, CA, USA). All data sets were checked for normality using a Shapiro–Wilk test. Significance in data comparisons was determined using parametric statistics if data passed normality (ANOVA: one-way analysis of variance, supplemented as needed with Students *t*-test); otherwise, we performed non-parametric statistics (ANOVA on ranks, Mann–Whitney or Welch’s test). We defined statistical significance as *p* ≤ 0.05.

## Results

### Collagen mimetic peptides improve tear film production and corneal sensitivity after desiccation stress

**FIGURE 2:**
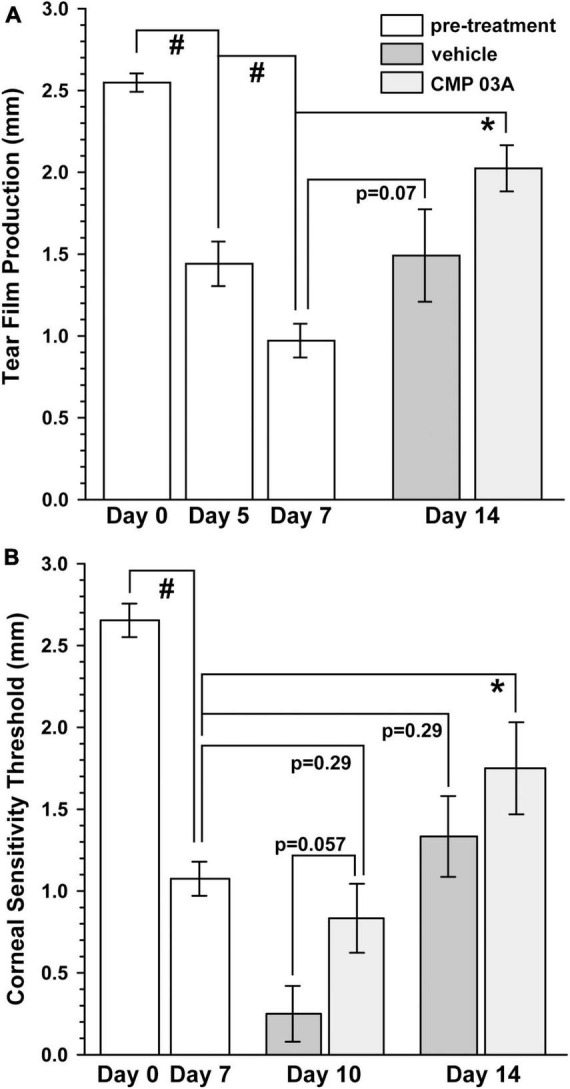
CMP 03A improves tear film production and corneal sensitivity. **(A)** The two-week desiccation regimen significantly reduced tear production in mice after 5 and 7 days (#, *p* < 0.001) compared to baseline (day 0). Tear film production did not improve in vehicle-treated mice (day 7 vs. day 14, *p* = 0.07), but did with CMP 03A treatment (day 7 vs. day 14; *, *p* < 0.001). **(B)** Corneal sensitivity was significantly reduced after 7 days of the desiccation regimen (#, *p* < 0.001). After 3 days of vehicle treatment, sensitivity was further reduced (day 10 vehicle vs. CMP 03A; *p* = 0.057), but not with CMP 03A (*p* = 0.29). On day 14 after 7 days, CMP 03A treatment improved sensitivity (*, *p* = 0.01), while vehicle did not (*p* = 0.29). *N* = 11–29 (vehicle), 11 (CMP 03A), and 13 (naïve).

Patients with dry eye disease exhibit corneal hypoesthesia in addition to depletion of tear film. The extent of the corneal epithelial nerve damage positively correlates with decreased corneal sensitivity (Bourcier et al., 2005). Similarly, murine models of dry eye disease have shown reduced overall corneal sensitivity (Stepp et al., 2018). Atropine and desiccation reduced tear film production by 43% after 5 days and by 60% after 7 days compared to baseline day 0 (*p* < 0.001, Figure 2A). Following 7 days of vehicle treatment, tear production remained low (day 14 vs. day 7, *p* = 0.07); CMP 03A significantly improved tear production (day 14 vs. day 7, *p* < 0.001). We also used a Cochet-Bonnet aesthesiometer to determine the effect of desiccation on central corneal nerve sensitivity. After 7 days, corneal sensitivity threshold declined by 60% compared to baseline (1.08 ± 0.10 mm vs. 2.65 mm ± 0.10 mm; *p* < 0.001, Figure 2B). By day 10, after 3 days of vehicle treatment, corneal sensitivity continued to decline (day 7 vs. day 10, *p* < 0.001); CMP 03A reversed this trend (day 10 vs. day 7, *p* = 0.29). By day 14, after 7 days of CMP, sensitivity improved compared to day 7 (*p* = 0.01), while vehicle cornea still demonstrated reduced sensitivity (*p* = 0.29).

### Desiccation-induced corneal nerve bed damage is prevented by collagen mimetic peptide treatment

**FIGURE 3:**
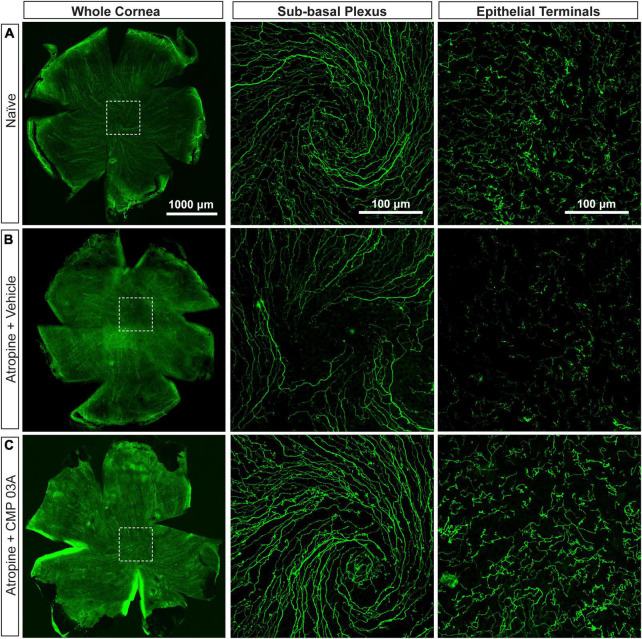
Central corneal nerve bed coverage in naïve, vehicle-, and CMP 03A-treated mice. Representative confocal images of βIII-tubulin-stained nerve fibers in the whole cornea with higher magnification images of the central sub-basal plexus and epithelial terminals. The dashed boxes indicate central swirl location. **(A)** Naïve animals have an intact central nerve fiber swirl and robust sub-basal plexus and nerve terminal coverage. **(B)** Desiccation with vehicle treatment only led to reduced sub-basal plexus and epithelial terminal coverage. **(C)** CMP 03A-treated mice did not exhibit a reduced nerve coverage at the sub-basal or terminal epithelial plexus.

To assess central nerve structure, we labeled corneas with antibodies to βIII-tubulin (Figure 3). In naïve mice, central corneal nerve fibers in the sub-basal epithelial plexus exhibit an intact characteristic swirl pattern (sub-basal plexus; Figure 3A). These nerve fibers branch out at a 90-degree angle in the anterior direction and continue to extend up between apical cells of the epithelium where nerve terminals are evident (epithelial terminals). Desiccation and vehicle treatment led to reduced neuronal coverage in the sub-basal plexus, as evidenced by a decrease in nerve fibers and loss of the intact central nerve fiber swirl (Figure 3B). Epithelial nerve terminals also reduced in density compared to naïve. CMP 03A significantly improved corneal nerve bed survival at both the sub-basal and epithelial terminal plexus when compared to the vehicle treated group (Figures 3C vs. 3B).

### Collagen mimetic peptide reduces nerve fragmentation

**FIGURE 4:**
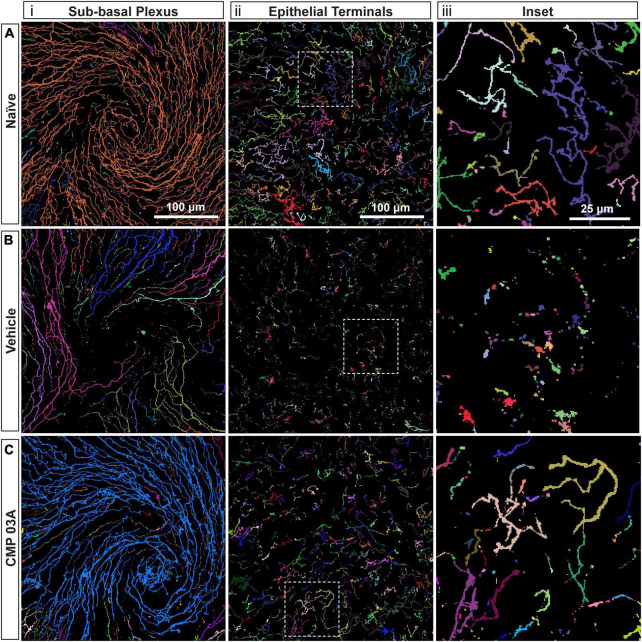
CMP reduces nerve fragmentation. Representative pseudo-colored nerve fragmentation images from **(A)** naïve, **(B)** desiccation + vehicle-, and **(C)** desiccation + CMP 03A-treated mice. Images show fragmentation in the **(i)** sub-basal plexus and **(ii)** epithelial terminals. Dashed boxes indicate location of enlarged inset images **(iii)**. Desiccation + vehicle increased nerve fragmentation as shown by the increase in frequency of distinctly colored fragments in the sub-basal plexus **(B,i)**. Although reduced in number, remaining epithelial terminals were more fragmented than in naïve and CMP 03A-treated mice (**B,ii**; inset). **(C)** CMP 03A-treated mice exhibited less nerve fragmentation in the sub-basal plexus **(C,i)** and in epithelial terminals **(C,ii)**. Scale bars as indicated.

To assess the extent of nerve degeneration, we quantified fragmentation of βIII-tubulin-labeled nerve fibers. The number of contiguous nerve fragments in each sub-basal and epithelial terminal image were determined. Representative pseudo-colored fragmentation images are shown in Figure 4, where each contiguous nerve fragment is identified by a unique color. In naïve mice, sub-basal nerve fibers in the central swirl region were formed by long, contiguous fragments (Figure 4Ai). Similarly, epithelial terminals, although numerous, were intact; each nerve terminal was mostly unicolored (Figure 4Aiii, inset). In the vehicle group, there were a larger number of shorter, contiguous nerve fragments at the sub-basal plexus (Figure 4Bi), suggesting discontinuity. Compared to naïve animals, epithelial nerve terminals were reduced in number and highly fragmented (Figures 4Bii, iii; inset), indicating extensive degeneration. Interestingly, CMP treatment reversed this trend; nerves appeared to have greater continuity and were much less fragmented at the sub-basal (Figure 4Ci) and epithelial levels (Figures 4Cii, iii; inset), and exhibited levels of fragmentation similar to naïve mice.

**FIGURE 5:**
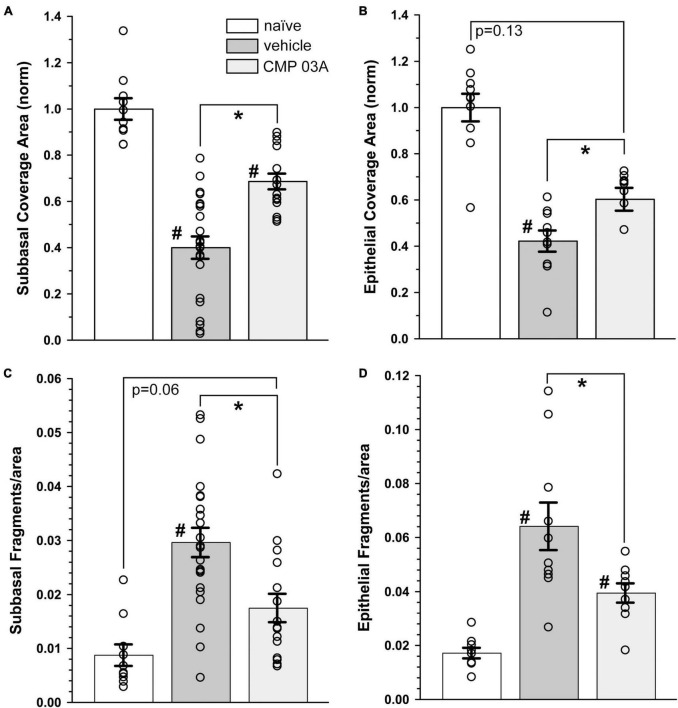
CMP 03A prevents desiccation-induced corneal nerve degeneration. **(A)** Desiccation regimen significantly reduced sub-basal nerve coverage in vehicle-treated mice compared to naïve (#, *p* < 0.001). CMP 03A improved coverage compared to vehicle (*p* = 0.001). **(B)** Vehicle significantly reduced epithelial coverage compared to naïve (#, *p* = 0.001). CMP 03A increased epithelial coverage compared to vehicle (*p* = 0.001) to a level that did not significantly differ from naïve (*p* = 0.13). **(C)** The desiccation regimen caused significantly higher sub-basal fragmentation in vehicle-treated mice compared to naïve (#, *p* < 0.001); CMP 03A reduced fragmentation (*, *p* < 0.001) similar to naïve levels (*p* = 0.06). **(D)** In the vehicle group, epithelial terminal fragmentation increased compared to naïve (#, *p* < 0.001); CMP 03A reduced this trend compared to vehicle (*, *p* = 0.02). Results obtained from imaging as described in Figures 3, 4 and replicated as follows: *n* = 10 (naïve), 22 and 10 (vehicle sub-basal and epithelial, respectively), and 15 and 9 (CMP 03A sub-basal and epithelial, respectively).

We next quantified central nerve area coverage and fragmentation of the sub-basal plexus and epithelial terminal layers. In the vehicle cohort, the two-week desiccation regimen reduced neuronal coverage of the sub-basal plexus by 60 ± 5% (*p* < 0.001; Figure 5A), and epithelial terminals by 58 ± 5% compared to naïve cornea (*p* < 0.001; Figure 5B), with some corneas in the vehicle group demonstrating completely degenerated nerve beds. CMP treatment expanded both sub-basal (+72%) and epithelial (+42%) neuronal coverage compared to vehicle treatment (*p* = 0.001). Accordingly, both sub-basal and epithelial terminals demonstrated a 3-fold increase in fragmentation, indicative of degenerative disassembly (*p* < 0.001; Figures 5C, D). Treatment with CMP significantly reduced fragmentation by 40% in both zones (*p* ≤ 0.02; Figures 5C, D). These results together suggest that topical CMP treatment preserves corneal nerve integrity during desiccating conditions.

**FIGURE 6:**
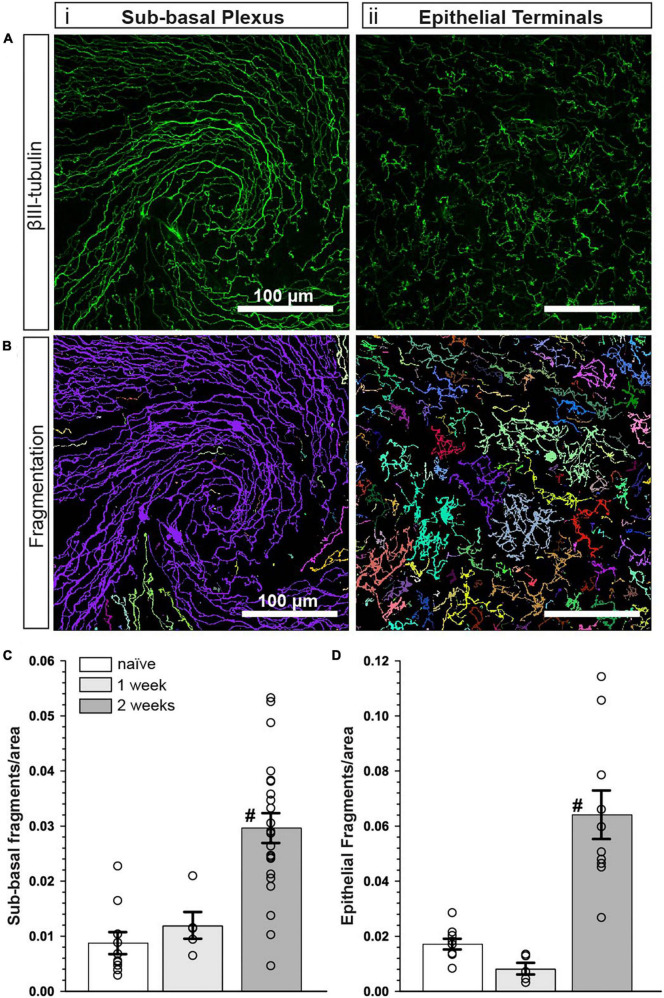
Corneal nerve bed is intact after 1 week of desiccation. **(A)** Representative confocal images of βIII-tubulin-stained nerve fibers in the **(i)** central sub-basal plexus and **(ii)** epithelial terminals after 1 week of desiccation. **(B)** Representative pseudo-colored nerve fragmentation images from the **(i)** central sub-basal plexus and **(ii)** epithelial terminals. **(C)** After 1 week, the desiccation regimen did not cause significant fragmentation in the sub-basal plexus compared to naïve (*p* = 0.60). **(D)** Similarly, there was no significant fragmentation of epithelial terminals (*p* = 0.30). Compared to 1 week, nerve fragmentation in both the sub-basal **(C)** and epithelial plexus **(D)** at 2 weeks with vehicle treatment was significantly increased (^#^, *p* ≤ 0. 01). Scale bars as indicated. Experiment replicated as in Figure 5 for naïve and vehicle (2 weeks); *n* = 5 (vehicle 1 week).

The influence of CMP treatment in our regimen could be either restorative to damage occurring during the first week or protective of damage during the second. Although tear film production and corneal sensitivity were diminished after 1 week (Figure 2), βIII-tubulin-labeled nerve fibers in both the sub-basal plexus and layer of epithelial terminals appeared no different than naïve, without apparent increases in fragmentation using our algorithm (Figures 6A, B). When quantified, fragmentation of neither the sub-basal plexus nor epithelial terminals differed significantly from naïve (Figures 6C, D; *p* ≥ 0.30). Compared with 1-week data, nerve fragmentation at 2 weeks showed significantly increased nerve degeneration in both the sub-basal and epithelial levels (Figures 6C, D; *p* ≤ 0.01). These results suggest that CMP treatment in this regimen acts to preserve nerve structure in a protective rather than restorative manner.

### Collagen mimetic peptide treatment preserves the integrity of the corneal epithelium

**FIGURE 7:**
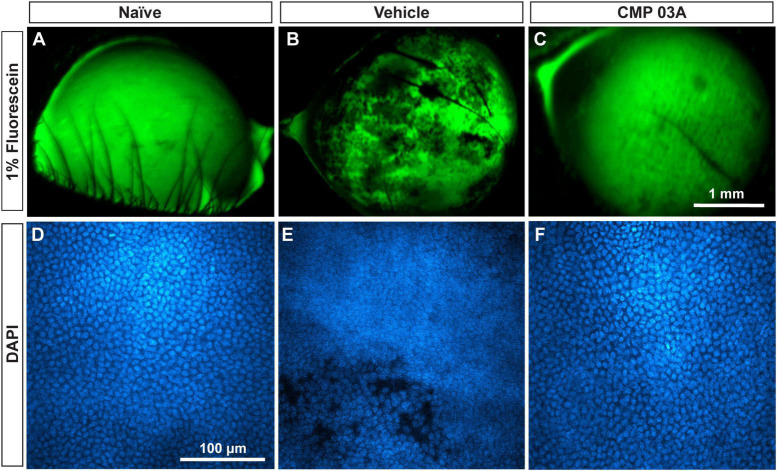
CMP 03A prevents desiccation-induced disruption of the corneal epithelium. Representative images of the corneal epithelial surface in **(A)** naïve, **(B)** vehicle- and **(C)** CMP-treated mice visualized *in vivo* by fluorescein staining. CMP 03A prevented corneal epithelial pitting observed in the vehicle group. Representative DAPI-stained confocal images of the epithelial layer in **(D)** naïve, **(E)** vehicle- and **(F)** CMP 03A-treated mice. Vehicle treatment caused epithelial disruption that is absent in the CMP 03A-treated cohort and comparable to naïve animals. Scale bars as indicated.

We determined *in vivo* how CMP treatment affected corneal epithelium with desiccation stress using sodium fluorescein staining (Figure 7). The surface of naïve eyes showed uniform staining with little to no imperfection (Figure 7A). Vehicle-treated cornea, however, exhibited uneven, pitted staining, indicative of epithelial damage (Figure 7B). CMP-treated animals did not exhibit as many imperfections and appeared similar to naïve animals (Figure 7C). These results suggest that the two-week desiccation regimen perturbs the cells of the corneal epithelium, a pathology prevented with topical CMP treatment.

Next, we assessed epithelial cell damage using DAPI-staining; representative images are shown in Figure 7. In naïve animals, the central epithelium was uniform, with clearly defined DAPI-stained nuclei (Figure 7D). In vehicle-treated animals, the integrity of the epithelium was diminished and definition between nuclei reduced with areas of epithelial cell loss, and also showed indications of localized damage (Figure 7E). However, CMP-treated mice resembled naïve animals; cell bodies were uniform and clearly defined with no evidence of damage (Figure 7F).

**FIGURE 8:**
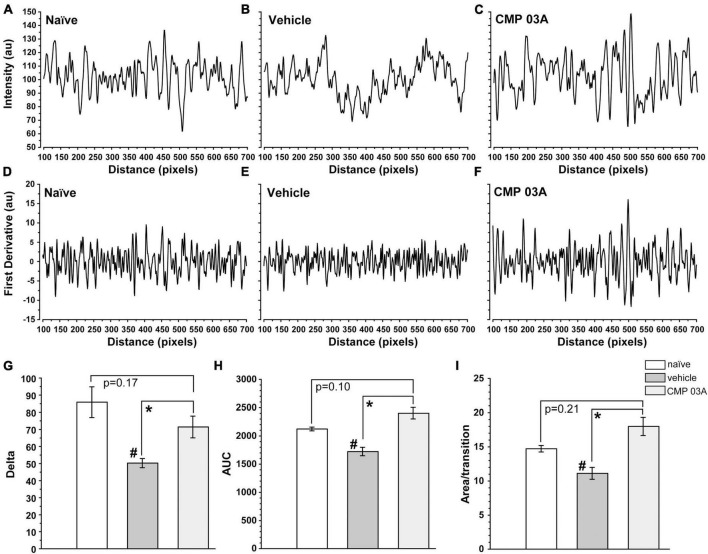
CMP 03A preserves the integrity of the corneal epithelial cell layer. Examples of fluorescence intensity and first-derivative line plots, respectively, in naïve **(A,D)**, vehicle **(B,E)**, and CMP-treated **(C,F)** mice. Desiccation reduces the amplitude of the derivative compared to naïve; CMP reverses this trend. **(G)** The transition delta is significantly reduced in the vehicle group compared to naive (#, *p* = 0.01), which is reversed by CMP 03A (*, *p* = 0.035). **(H)** The area under the curve (AUC) is significantly reduced by vehicle compared to naïve (#, *p* = 0.002); CMP 03A improves this trend compared to vehicle (*, *p* < 0.001), elevating to naïve levels. Similarly, the area under the curve per transition **(I)** is significantly reduced in vehicle compared to naïve (#, *p* = 0.008). CMP 03A improves this compared to vehicle (*p* = 0.001), once again to naïve levels. Results obtained from imaging as described in Figure 7 and replicated as follows: *n* = 3 (naïve), 6 (vehicle), and 8 (CMP 03A).

To further assess the integrity of the epithelium we analyzed the distribution of DAPI intensity in the central cornea. By drawing multiple fluorescence intensity line plots across each image, we extracted information regarding the uniformity and periodicity of DAPI-stained nuclei characteristic of naïve epithelium (Figure 8A). Intensity across space for naïve cornea shows regular intensity fluctuations, indicative of the pattern of higher intensity DAPI nuclei and lower intensity background in transitions between cells. In vehicle-treated animals, the amplitude of intensity fluctuations and their regularity were reduced (Figure 8B). However, CMP treatment increased amplitude and regularity, similar to naïve cornea (Figure 8C).

To better resolve the pattern of transition in DAPI staining, we calculated the first derivative of the intensity line plots (Figures 8D–F). A peak in the derivative corresponds to a transition to higher fluorescence (i.e., a cell nucleus), whereas a trough represents the transition from cell nucleus to background (i.e., the nucleus edge). The area under the curve of the derivative (AUC) gives the amplitude of the intensity of transitions in each group. Thus, a sample with clearly defined, high intensity epithelial nuclei will have large intensity transitions (larger AUC) compared to a sample with more ambiguous transitions (lower AUC). In vehicle-treated animals, the amplitude of transitions on the first derivative plot was reduced compared to naïve animals (Figure 8E); this trend was countered by CMP 03A treatment (Figure 8F). The absolute value of the difference in amplitude between peaks and troughs in the first derivative (the delta) was significantly lower in the vehicle group compared to naïve (*p* = 0.01, Figure 8G); CMP improved this outcome compared to vehicle (*p* = 0.035). Next, we calculated the combined AUC of peaks and troughs (Figure 8H) and the area apportioned to each transition in the derivative (Figure 8I). Vehicle-treated animals had a significantly lower AUC than naïve (*p* = 0.002) and CMP animals (*p* < 0.001). In the vehicle-treated group, the area per transition was reduced vs. naïve (*p* = 0.008); this was countered by CMP 03A (*p* = 0.001). By all three measures, CMP 03A restored epithelial integrity to naïve levels. Taken together, these data suggest that while desiccation effectively disrupts the epithelial surface, topical CMP treatment acts to preserve the integrity of the epithelium.

## Discussion

The densely innervated nerve bed of the cornea is a critical component in maintaining the homeostatic function of the ocular surface (Vereertbrugghen and Galletti, 2022). Corneal nerves aid in controlling tear film production that not only prevents surface desiccation, but also contributes to immune regulation of the ocular surface and to the integrity of the corneal epithelial layer (Galletti and de Paiva, 2021). In ocular surface diseases such as dry eye, damage to the corneal surface (including collagen itself) leads to a perpetual cycle of epithelial damage, increased inflammation, and progressive nerve degeneration (Beuerman and Schimmelpfennig, 1980). The cornea is a highly collagenous structure and damage to collagen has been strongly associated with age-related increases in susceptibility to injury or disease (Frantz et al., 2010; Sandhu et al., 2012). As such, targeting collagen to promote corneal healing may present a novel therapeutic avenue for the treatment of numerous ocular surface diseases. Our recent work demonstrated that CMPs show great promise in promoting corneal cellular integrity and function after acute injury (Baratta et al., 2022). Our results herein further this work and indicate that CMPs may also act to preserve corneal nerve bed structure and function during desiccating conditions.

In this study we combined pharmaceutical (atropine) and mechanical (air turbulence) desiccation to create a mouse model of ocular surface desiccation that recapitulates many of the features of dry eye disease. The two-week regimen effectively reduced tear film production (Figures 1, 2A). In addition, central corneal sensitivity was significantly diminished after 7 days of desiccation and even further by day 10 in the vehicle-only group (Figure 2B). Our results show that topical treatment with CMP for just 1 week during desiccation improved tear film production and partially restored corneal sensitivity (Figure 2).

Diminished corneal sensitivity is indicative of degenerative damage to the corneal nerve bed (Stepp et al., 2018), thus we visualized central corneal nerves with antibodies targeted to βIII-tubulin (Figure 3). Two weeks of desiccation in the vehicle-only group led to a reduction in the number of corneal sensory nerves and their terminals that extend apically into the epithelium. This loss presumably contributed to our observation of decreased corneal sensitivity (Figure 2B). Interestingly, mice that received topical CMP treatment during desiccation showed evidence of significant nerve bed preservation in both the sub-basal and terminal plexus (Figure 3) and had a significantly improved sensitivity threshold (Figure 2B), suggesting functional recovery. Fragmentation of peripheral nerve fibers such as those found in the cornea is an early feature of the axonal degeneration process (Cajal, 1928; Coleman and Hoke, 2020). In addition to expanding corneal nerve coverage, our results show that CMP treatment reduced the level of nerve fragmentation, which is indicative of degenerative assembly of neurons (Figure 4). This striking preservation of corneal nerve bed may be a consequence of damaged collagen repair by CMP which can impact both the structure and stability of nerves and the integrity of the epithelium through improvement of the extracellular matrix microenvironment.

Since CMP treatment preserved nerve integrity in our model, we sought to determine whether the actions of CMP were neuroprotective or restorative in nature. Corneal samples at day 7 did not show signs of nerve degeneration (Figure 6), suggesting that CMP preserved, rather than restored nerve structure. Our results indicate that tear film and sensitivity measures were not fully restored to baseline levels (Figure 2), which suggests that the neuroprotective effect of CMP in this setting is tear-film independent. Since CMPs have a high affinity for collagen fragments, we hypothesize that the preservation of collagen structure may be facilitating the neuroprotective effects we observe. However, future studies testing the direct effects of CMP on collagen bioactivity and corneal nerve function are required to understand fully the mechanism of CMP action.

A mutual trophic support system exists in the avascular cornea whereby the health of nerves is supported by cells of the epithelium and vice versa (Labetoulle et al., 2019). The corneal epithelium shows signs of impairment and damage in patients with acute and chronic DED (Perez et al., 2020). While corneal nerves can stimulate epithelial cell differentiation, growth, proliferation, and the production of collagen through the release of neuropeptides and neurotrophins (Baker et al., 1993), epithelial cells can directly impact neuronal survival and growth through the release of trophic factors such as nerve growth factor (NGF) (Emoto and Beuerman, 1987). Application of topical CMP prevented disruption of the epithelium that occurred during desiccation (Figures 7, 8). By directly annealing to damaged collagen fragments, the effects of CMP in our model may be attributed to the repair of corneal collagen. This repair in turn can promote epithelial integrity and proliferation to recover epithelium damaged by desiccation stress (Baratta et al., 2021, 2022). Alternatively, preserving helical collagen structure is an important prerequisite in promoting homeostatic cell signaling (San Antonio et al., 2020). By repairing breaks in collagen strands that may trigger inflammation, CMPs may act to reduce local inflammation and further damage.

Our results overall suggest that CMP is multifaceted in its protection of the corneal surface during desiccation stress, acting to preserve central nerve bed structure and to improve epithelial integrity. CMPs may therefore show promise in promoting the health and function of nerves in the cornea in the treatment of dry eye disease.

## Data availability statement

The raw data supporting the conclusions of this article will be made available by the authors, without undue reservation.

## Ethics statement

This animal study was reviewed and approved by the Vanderbilt University Institutional Animal Care and Use Committee.

## Author contributions

RB, BD, ES, LW, and DC conceived of the study. LW and DC designed the research and prepared the manuscript. LW, JH, and OB performed research. LW, JH, and DC analyzed data. All authors contributed to the article and approved the submitted version.
